# Association Between Biochemical Parameters, Parathyroid Gland Morphometry, and Dual-Phase MIBI SPECT/CT Findings in Primary Hyperparathyroidism

**DOI:** 10.3390/jcm15051973

**Published:** 2026-03-04

**Authors:** Anna Krzentowska, Aleksander Józef Konturek, Filip Gołkowski, Anna Merklinger-Gruchała, Marcin Barczyński

**Affiliations:** 1 Department of Endocrinology and Internal Medicine, Andrzej Frycz Modrzewski Krakow University Medical College, 30-705 Kraków, Poland; fgolkowski@uafm.edu.pl; 2 Department of Endocrine Surgery, Faculty of Medicine, Jagiellonian University Medical College, 31-501 Kraków, Poland; aleksander.konturek@uj.edu.pl (A.J.K.); marcin.barczynski@uj.edu.pl (M.B.); 3 Department of Bioinformatics and Public Health, Andrzej Frycz Modrzewski Krakow University Medical College, 30-705 Kraków, Poland; amerklinger-gruchala@uafm.edu.pl

**Keywords:** primary hyperparathyroidism, 99m-Technetium-Sestamibi, PTH, calcium

## Abstract

**Objective**: This study assessed the relationship between serum calcium (Ca) and parathyroid hormone (PTH) levels, the size and weight of the enlarged parathyroid gland (PG), and the results of technetium-99m-metoxyisobutylisonitrile (MIBI) parathyroid scintigraphy with single-photon emission computed tomography/computed tomography. **Methods**: Among 252 patients who underwent surgery for hyperparathyroidism between October 2022 and March 2025 at the Department of Endocrine Surgery of the University Hospital in Krakow, 212 patients with primary hyperparathyroidism (PHPT) were selected and divided into the MIBI(−) (n = 49) and MIBI(+) (n = 163) groups. **Results**: MIBI was positive in 76.9% and negative in 23.1% patients with PHPT. Mean PTH in the MIBI(+) and MIBI(−) groups was 177.3 ± 144.7 and 127.7 ± 59.4 pg/mL, respectively. Significant differences were found in PTH (*p* < 0.01), maximum excised PG size (*p* < 0.01), and weight (*p* < 0.01). PTH predicted positive scintigraphy in univariate analysis (OR = 2.65; 95% CI: 1.19–5.89; *p* = 0.02) and showed a borderline association in the multivariate model (OR = 2.47; 95% CI: 0.95–6.41; *p* = 0.06). Optimal cut-offs for predicting MIBI positivity were 135.0 pg/mL for PTH (AUC = 0.64), 0.73 g for PG weight (AUC = 0.81), and 18.0 mm for PG maximum size (AUC = 0.78). The Wisconsin index (Ca × PTH) was associated with positive MIBI (OR = 2.58; 95% CI: 1.15–5.78; *p* = 0.02), with an optimal cut-off of 256.0. Serum total Ca levels showed no significant association with positive MIBI (*p* = 0.60). **Conclusions**: Serum PTH levels, Wisconsin index, and enlarged PG size and weight may help predict parathyroid scintigraphy outcomes.

## 1. Introduction

Primary hyperparathyroidism (PHPT) is an endocrine disease characterized by elevated serum total Ca and PTH levels along with hypophosphatemia [1]. The most common cause of PHPT is a solitary parathyroid adenoma (PA), followed by multiglandular disease, including double adenomas or parathyroid hyperplasia (PH), and then parathyroid carcinoma (PC) as the least common cause [2,3]. Currently, the World Health Organization classification of parathyroid tumors from 2022 is being utilized [4].

Although surgery, which involves the removal of the affected parathyroid gland (PG), remains the primary treatment approach for PHPT [5,6,7], surgical approaches have undergone significant changes, starting with bilateral neck exploration and ending with the currently preferred, much more precise minimally invasive parathyroidectomy (MIP) [8]. However, the effectiveness of MIP depends on the correct location of the affected PGs. Ultrasound (US), technetium-99m-metoxyisobutylisonitrile (99mTc-MIBI) scintigraphy, and computed tomography (CT) are commonly used imaging modalities for the diagnosis of PHPT [9,10]. Dual-phase MIBI single-photon emission computed tomography (SPECT) operates on the principle that the tracer washes out more rapidly from the thyroid gland than from the abnormal parathyroid tissue, allowing for delayed imaging to highlight PAs [5,11]. Additional imaging modalities used for the diagnosis of PHPT are magnetic resonance imaging (MRI), four-dimensional CT, 18F-fluorocholine positron emission tomography/CT (18F-FCH PET/CT), and minimally invasive radio-guided parathyroidectomy [12,13]. Despite biochemical confirmation of PHPT, accurate visualization of abnormal PGs remains challenging.

Several studies have examined the correlation between various biochemical parameters and scintigraphy results to determine which have the greatest effect [14,15,16,17,18]. Accordingly, cut-off points for serum total Ca and intact PTH (iPTH) concentration have been explored. Studies have shown that at an iPTH level of 150 pg/mL [14], 124.5 ng/L [15], or 79.4 ng/L [19], positive MIBI scintigraphy results were obtained. Notably, statistically significant differences in iPTH levels and PA size were found between patients with positive and negative MIBI scintigraphy results [15,20,21]. Studies regarding total Ca have provided variable data, with many showing no relationship between total Ca levels and scintigraphy results [15,21,22]. Meanwhile, the relationship between other biochemical parameters and scintigraphy results has also been studied [15,19].

The identification of PAs is crucial for obtaining optimal results following MIP. Evidence has shown that 18F-choline PET/CT has a higher sensitivity and specificity than conventional scintigraphy methods [23,24,25,26,27,28]. Intraoperative PTH examination also plays an important role in the localization of altered PGs [29,30]. Moreover, sensitivity in differentiating between PA and PH has been investigated [31].

Considering all the aforementioned issues, we attempted to assess the impact of various biochemical and clinical parameters on parathyroid scintigraphy results in a cohort of patients with PHPT. Accordingly, the primary objective of the current study was to evaluate the correlation between 99mTc-MIBI SPECT/CT results and biochemical factors (serum total Ca and PTH) and PG size and weight. The secondary objective of this study was to evaluate the cut-off points at which the aforementioned parameters would be predictive of positive parathyroid scintigraphy.

## 2. Materials and Methods

### 2.1. Characteristics of the Study Group

Data from 252 patients with hyperparathyroidism (HPT) who underwent surgery at the Department of Endocrine Surgery of the Jagiellonian University Medical College at the University Hospital in Krakow between 10 October 2022 and 31 March 2025 were retrospectively analyzed. Among them, 32 patients who underwent surgery for secondary or tertiary hyperparathyroidism were excluded. Moreover, 220 patients had PHPT, whereas 6 patients with PHPT did not undergo scintigraphy. In two patients, histopathological examination revealed no pathological change in the parathyroid glands. Ultimately, 212 patients were included for analysis and divided into two groups: the negative scintigraphy group and the positive scintigraphy group. Positive scintigraphy was defined as visible tracer uptake, whereas negative scintigraphy was defined as the absence of evident uptake. Among the included patients, 7 underwent a magnetic resonance imaging (MRI) of the neck, whereas 10 patients underwent (18)F-choline PET/CT. Demographic information, preoperative and postoperative laboratory results, preoperative imaging findings, surgical approaches, and pathological reports were recorded. Patient data were anonymized and collected in the international EUROCRINE database [32].

### 2.2. Diagnosis

**Figure 1:**
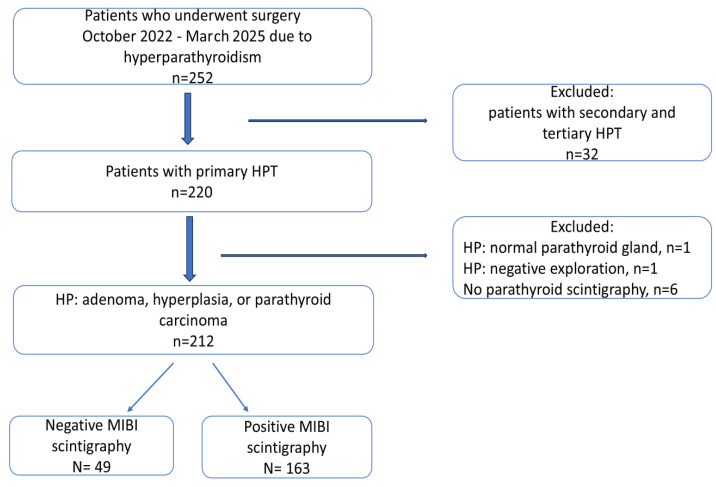
Flowchart showing the inclusion and exclusion processes.

Serum total Ca and PTH levels were measured before surgery for biochemical confirmation of PHPT. The normal reference ranges for serum total Ca, serum ionized Ca, and PTH were as follows: 2.20–2.55 mmol/L, 1.12–1.32 mmol/L, and 15.00–56.90 pg/mL, respectively. Patients were referred for surgery if they had biochemically confirmed PHPT (elevated serum PTH and Ca levels; in a few cases, normocalcemia was observed, which was due to prior pharmacological treatment). Hypercalcemia and elevated PTH levels were noted in 115 patients. In five patients, normocalcemia was observed despite elevated PTH levels. To identify the cause of the disease, patients underwent US of the thyroid and PGs, 99-Technetium MIBI parathyroid scintigraphy, and MRI of the neck and 18-F-choline PET/CT in individual cases. Biochemical tests (Ca and PTH), US, and scintigraphy of the PGs were performed at various clinical centers before admission to the surgery department. After the surgery, tissue samples were histopathologically (HP) examined. Serum total Ca and PTH levels were assessed on the first day after surgery. The morphometric parameters (weight and size) of the excised PG were assessed. A total of 212 eligible patients were included in the analysis for factors influencing the results of parathyroid scintigraphy. A visual representation of this selection process is shown in Figure 1.

### 2.3. Performance Characteristics of Technetium-99m-Metoxyisobutylisonitrile (MIBI) Parathyroid Scintigraphy with Single-Photon Emission Computed Tomography/Computed Tomography

All examinations were performed using a SPECT/CT gamma camera system (Symbia T16, Siemens Healthcare, Hoffman Estates, Illinois (IL), USA). Following intravenous administration of 500–550 MBq of Tc-99m Sestamibi (MIBI) in adult patients, a dual-phase imaging protocol was applied. The protocol is based on the differential washout kinetics of Tc-99m MIBI, which clears more rapidly from normal thyroid parenchyma than from hyperfunctioning parathyroid tissue. Patients were positioned supine during image acquisition. Planar images were acquired in the anterior projection, encompassing the neck and mediastinum. Low-energy high-resolution (LEHR) collimators were used with a 140 keV energy window (±15%) and a 128 × 128 acquisition matrix. Early-phase imaging was performed 15–20 min after tracer injection, followed by delayed-phase imaging at approximately 2 h post-injection. Each projection was acquired for 10 min or until 500,000 counts were obtained. Subsequently, SPECT acquisition was performed after the delayed phase using identical collimators and acquisition parameters. A 180° detector rotation was applied with the dual-head gamma camera system. CT acquisition was performed in all patients using the integrated SPECT/CT system with the following parameters: tube voltage 130 kVp, automatic tube current modulation, slice thickness 2 mm, and a standard soft-tissue reconstruction algorithm. CT data were used for anatomical localization and attenuation correction. For image interpretation, a parathyroid adenoma was defined as a focal area of increased tracer uptake persisting on delayed images. In cases of diffuse parathyroid hyperplasia, uptake was typically less focal and more diffuse. Potential artifacts included conditions affecting thyroid tracer retention, particularly thyroid nodules with delayed washout, which may result in false-positive findings.

### 2.4. Statistical Analysis

Continuous variables were reported as means ± standard deviation (SD) and as medians with interquartile ranges (Q1–Q3), whereas categorical variables were reported as numbers (n, %). Comparisons between groups (positive vs. negative SPECT/CT; adenoma vs. hyperplasia) were performed using the chi-square/Fisher’s exact tests for categorical variables and the Mann–Whitney U test for continuous variables. Pre- vs. postoperative PTH and Ca levels were assessed using the Wilcoxon signed-rank test. Multivariable logistic regression was conducted to identify predictors of positive scintigraphy. Continuous predictors (i.e., age, Ca, PTH, and gland weight/size) were standardized (z-scores) for effect size comparison.

To evaluate the relationship between total Ca and PTH levels and the likelihood of a positive scintigraphy result, a moving window of 15 consecutive patients was used to define a diagnostic range for each predictor. Patients were classified as within or outside this range, and odds ratios (ORs) for positive scintigraphy were calculated. Smoothed risk profiles were generated using LOESS regression (smoothing parameter 0.25) to visualize the relationship between predictor values and positive scintigraphy probability. A two-dimensional grid search was performed to analyze 221 threshold combinations (13 Ca × 17 PTH cut-offs) to assess their combined predictive value, calculating the proportion of positive scintigraphy results for patients exceeding both thresholds. A heat map with a grayscale gradient highlighted the threshold combinations linked to higher probabilities. The product of total Ca (mmol/L) and PTH (pg/mL) (Wisconsin index) was included in the logistic regression to assess its association with MIBI positivity. All analyses were conducted using Statistica 13.3 (StatSoft Inc., Tulsa, OK, USA), with *p* < 0.05 indicating statistical significance.

## 3. Results

### 3.1. Characteristics of All Patients Who Underwent Surgery for PHPT (N = 220)

Among the 220 patients enrolled, 31 were men (14.1%), and 189 were women (85.9%). The median PTH was 134.0 (102.5–181.0) pg/mL, whereas the median total Ca level was 2.8 (2.7–2.9) mmol/L. A total of 180 patients (81.8%) achieved Ca normalization on the first postoperative day, whereas 21 patients (9.5%) failed to do so despite normalization of PTH. This failure may be due to our assessment of Ca on the first day after surgery, whereas patients with high preoperative serum Ca levels need 2–3 days postoperatively to reach a reference range. The results are presented in Table 1.

In 10 patients, F-18 choline PET/CT imaging was performed (positive in 8 patients and negative in 2 patients). This examination was carried out in patients with biochemically confirmed hyperparathyroidism in whom scintigraphy failed to demonstrate tracer uptake (negative result—6 cases). Two patients had not undergone scintigraphy, while in the remaining two patients, scintigraphy was positive. In the cases with negative F-18 choline PET/CT results, the parameters were as follows: in the first case, PTH, 118 pg/mL, Ca, 2.65 mmol/L, maximum PG dimension, 3 mm; in the second case, PTH, 174 pg/mL, Ca, 3.0 mmol/L, maximum PG dimension, 11 mm. Histological examination confirmed the presence of a PA in 9 cases and a PH in 1 case.

### 3.2. Analysis of Patients with Positive and Negative Parathyroid 99mTc MIBI (N = 212)

#### 3.2.1. Univariate Analysis of Parameters in the Negative and Positive MIBI Groups

A significant difference in preoperative serum level of PTH (*p* < 0.01), maximum dimension (*p* < 0.01), Wisconsin index (*p* < 0.01), and weight of the removed PG (*p* < 0.01) was noted between the MIBI(−) and MIBI(+) groups. However, no significant differences in total and ionized Ca levels were observed between the groups (*p* = 0.59 and 0.59, respectively). Also, the US results were significantly more positive in cases with a concomitant positive MIBI (*p* = 0.03). PAs were more frequent in the lower PGs (n = 137) than in the upper ones (n = 44), although the difference was not significant (Table 2).

Notably, one case of PC involved a 70-year-old woman (total preoperative Ca, 2.66 mmol/L; PTH, 179.0 pg/mL; negative MIBI SPECT/CT; positive US).

#### 3.2.2. Analysis of the Cut-Off Points for Total Ca, PTH, and the Max Size and Weight of the PG

**Figure 2:**
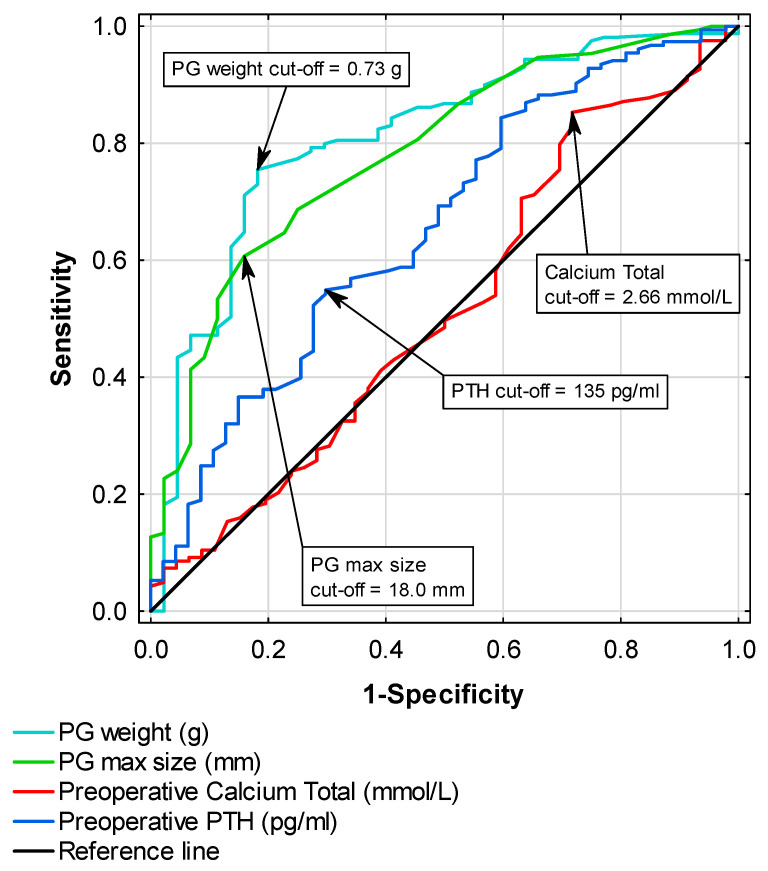
ROC curves showing the capacity of total preoperative serum Ca and PTH, PG maximum size, and total excised PG weight for predicting positive parathyroid scintigraphy. Optimal cut-off values determined by Youden’s index: 2.66 mmol/L for calcium (AUC = 0.53; 95% CI: 0.43–0.62), 135 pg/mL for PTH (AUC = 0.64; 95% CI: 0.55–0.74), 18 mm for PG max size (AUC = 0.78; 95% CI: 0.71–0.86), and 0.73 g for PG weight (AUC = 0.81; 95% CI: 0.74–0.88).

Receiver operating characteristic (ROC) curve analysis was performed to determine cut-off points for serum total Ca and PTH levels and PG maximum size and weight, assuming a positive result of parathyroid scintigraphy as the outcome. Youden’s method was used to determine the cut-off points, which were then placed on a chart (Figure 2).

The optimal cut-off for total Ca was 2.66 mmol/L, yielding an area under the curve (AUC) of 0.53 (95% confidence interval [CI]: 0.43–0.62). Despite high sensitivity (85.2%), the very low specificity (28.3%) resulted in frequent false positives. PTH performed better than Ca, with a cut-off of 135 pg/mL (AUC 0.64, 95% CI: 0.55–0.74), showing moderate sensitivity (55.3%) and higher specificity (68.1%). However, the difference in AUCs did not reach significance (*p* = 0.06). Much stronger discriminatory ability was observed for PG characteristics. In particular, weight (0.73 g) achieved an AUC of 0.81 (95% CI: 0.74–0.88), with sensitivity and specificity of 75% and 82%, respectively, whereas maximum size (18.0 mm) reached an AUC of 0.78 (95% CI: 0.71–0.86), combining moderate sensitivity (61%) with high specificity (84%). This finding indicated that weight provided better diagnostic value than did size due to its higher sensitivity at comparable specificity.

#### 3.2.3. Multivariate Analysis of the Impact of Biochemical Parameters Before Surgery on Positive Scintigraphy

Univariate (crude) logistic regression analysis showed that preoperative PTH levels were significantly associated with the likelihood of a positive scintigraphy (OR = 2.65; 95% CI: 1.19–5.89; *p* = 0.02). This association remained significant after adjustment for Ca levels in multivariate model 1. However, after additional adjustment for maximum PG diameter in model 2, the effect size remained similar (OR = 2.47; 95% CI: 0.95–6.41), although the association no longer reached statistical significance. This suggests that the relationship between PTH and MIBI positivity may be partially explained by tumor size, while still indicating a clinically relevant independent contribution of PTH. (Table 3).

Serum total Ca levels showed no significant association with positive scintigraphy in either the crude (OR = 1.15; 95% CI: 0.82–1.62; *p* = 0.40) or adjusted models (fully adjusted OR = 0.89; 95% CI 0.58–1.37); *p* = 0.60), indicating that this biomarker had no clear independent effect.

#### 3.2.4. Analysis of Positive Scintigraphy Risk Profiles for Both Quantitative Variables (i.e., Total Ca and PTH)

**Figure 3:**
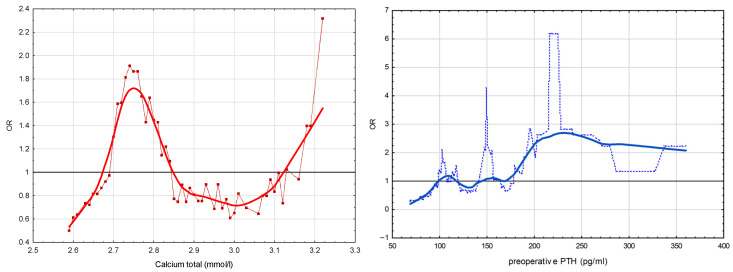
Smoothed risk profiles for PTH (blue line) and total Calcium (red line), using LOESS regression (smoothing parameter 0.25), with OR for positive scintigraphy.

Risk profiles for positive scintigraphy based on the values of both predictors were then analyzed. Smoothed risk profiles demonstrated a nonlinear relationship between each predictor and the likelihood of a positive scintigraphy. Higher preoperative PTH values were associated with an increased probability of a positive scintigraphy, with the risk beginning to constantly rise at approximately 135 pg/mL (OR above 1). The curves were generated using LOESS smoothing (span = 0.25), allowing visualization of gradual risk transitions across the full range of observed values (Figure 3, double graph of risk profiles).

Next, we tested whether simultaneous exceedance of both thresholds predicted positive scintigraphy. To this end, a combined analysis using grid search and heat map visualization was performed. PTH and Ca thresholds ranged from 90 to 170 pg/mL (5-unit steps) and 2.60 to 3.20 mmol/L (0.05 mmol/L steps), respectively, yielding 221 combinations. For each pair, the proportion of patients with positive scintigraphy among those exceeding both cut-offs was calculated and displayed in a heat map (Table 4), highlighting thresholds linked to more positive scintigraphy results.

The heat map demonstrated that the highest proportions of positive results were concentrated in areas where both Ca and PTH values were simultaneously elevated. This pattern suggested that the combined biochemical burden of these parameters, rather than their isolated values, might be relevant for scintigraphic detectability. Therefore, to formally quantify this observation, we examined whether the product of Ca and PTH (Wisconsin index) could serve as a single composite predictor of MIBI positivity. Univariate logistic regression analysis showed that each 1 SD increase in the Wisconsin index was associated with a 2.58-fold higher odds of positive scintigraphy (OR = 2.58; 95% CI: 1.15–5.78; *p* = 0.02).

**Figure 4:**
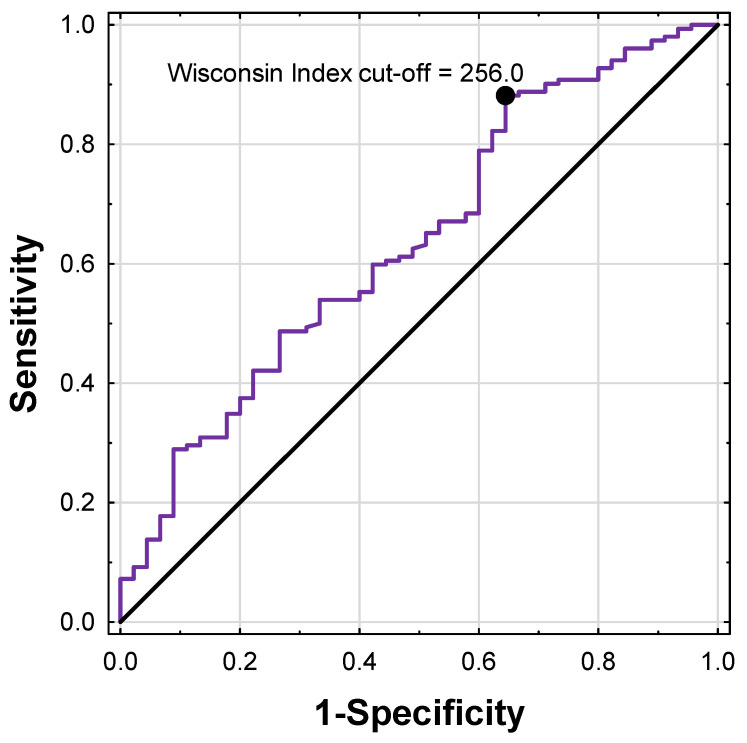
Receiver operating characteristic (ROC) curve illustrating the discriminatory ability of the product of preoperative total serum Ca (mmol/L) and PTH (pg/mL) (Wisconsin index) for predicting positive parathyroid scintigraphy. The optimal cut-off value determined using Youden’s index was 256.0 (AUC = 0.63; 95% CI: 0.55–0.73).

Receiver operating characteristic (ROC) curve analysis was performed to determine the optimal cut-off value of the Wisconsin index for predicting positive parathyroid scintigraphy, using Youden’s index (Figure 4). The optimal threshold was 256.0, yielding an area under the curve (AUC) of 0.63 (95% CI: 0.55–0.73). At this cut-off, sensitivity was 88.2%, and specificity was 35.6%, indicating high sensitivity but limited specificity. The discriminatory performance of the Wisconsin index was comparable to that of PTH alone (AUC 0.63 vs. 0.64), suggesting that the index does not substantially improve overall prediction but reflects a highly sensitive biochemical profile associated with MIBI positivity.

### 3.3. Analysis on the Efficacy of Surgical Treatment for PHPT in Patients with Negative MIBI (N = 49)

Although scintigraphy failed to detect altered PGs in 49 of the 212 patients (23.1%), all patients demonstrated significant biochemical improvement after surgery, with histopathology confirming pathological changes in each case (Table 5).

## 4. Discussion

Despite the importance of effective imaging for optimal surgical planning in PHP, this study showed that scintigraphy may fail to detect diseased glands in some cases. In our study, 76.9% and 23.1% of the patients had positive and negative MIBI, respectively, similar to the findings reported by Cordes et al. (73% and 27%, respectively) [14]. According to the literature, the uptake of the 99mTc-MIBI tracer can be influenced by PTH levels before surgery, as well as PA size and volume [15,20,21,33], vascularity, cystic changes, and tumor composition [11,34]. Mitochondria play a special role, considering that lipophilic 99mTc-MIBI accumulates therein [11,35]. Given that PA cells, especially oxyphil cells, contain a large number of mitochondria, they exhibit increased tracer uptake. Parathyroid oxyphil cells are large, eosinophilic cells found in both adenomas and normal parathyroid tissue, with a large nucleus and abundant cytoplasm containing mitochondria crucial for the accumulation of the 99mTc-MIBI tag. They produce PTH to a lesser extent than the main cells, which are basophilic. Evidence suggests that positive scintigraphy is characterized by a higher percentage of oxyphilic cells [22].

Our study showed that higher preoperative PTH levels were associated with a greater likelihood of positive parathyroid scintigraphy. Although this association lost statistical significance after adjustment for adenoma size, the consistent effect size across models suggests that PTH remains an important contributor to MIBI positivity, in line with previous reports [15,20,21,33,36]. Given that typical PAs have a considerable number of mitochondria, the tracer accumulates in them. Increased adenoma activity during PTH synthesis is associated with an increased number of mitochondria and thus increased marker accumulation. This finding may explain the lack of evident tracer uptake in cases with slightly elevated PTH values. The study by Durmus et al. confirmed the impact of cellular composition on the results of parathyroid scintigraphy [33]. In our study, the cut-off point for PTH was 135.0 pg/mL, which is also similar to those obtained in other studies (150 pg/mL [14], 160 pg/mL [37], 124.5 ng/L [15], or 79.4 ng/L [19]). However, some studies have indicated higher mean PTH values in patients with negative scintigraphy results (mean PTH, 319.38 pg/mL) [38]. In clinical endocrinology practice, it is common to encounter patients with biochemically confirmed PHPT who have only mildly elevated serum PTH and Ca levels, yet present with negative scintigraphy results. Despite the lack of radiotracer uptake, the disease is present and, if untreated, carries the risk of significant complications. This highlights the potential utility of establishing threshold values, particularly for PTH, to support clinicians in further patient management. In our study, we also assessed the Wisconsin index to determine whether this parameter could provide additional information regarding the likelihood of obtaining a positive scintigraphy result. Ultimately, we found that two preoperative parameters—PTH and PG size—may help predict positive scintigraphy outcomes. These findings are clinically relevant, as they indicate that below these threshold values, scintigraphy may fail to show radiotracer uptake, which, however, does not exclude the presence of disease. Awareness of this phenomenon may help guide further diagnostic and therapeutic decision-making.

This study showed no significant differences in the concentration of total and ionized Ca between the MIBI(+) and MIBI(−) groups, which is consistent with the data from the literature [19,21,22,39]. Nevertheless, certain studies have indicated such a relationship [37]. Calcium and phosphate metabolism is not only influenced by many factors, mainly PTH, but also by the concentration of the active vitamin D, albumin concentration, acid–base balance, kidney function, or medication consumption (e.g., glucocorticoids or lithium). Low albumin levels in kidney and liver diseases or malnutrition can lower Ca levels in the blood. Furthermore, the active form of vitamin D also plays a role in lowering Ca levels, with a deficiency in vitamin D potentially masking the true severity of hypercalcemia. In Poland, many patients have vitamin D deficiencies, which could also affect the final concentration of serum Ca. In the case of hyperparathyroidism, the autonomous secretion of PTH can be held responsible for hypercalcemia, but the influence of other factors on the final blood Ca concentration should be considered. Moreover, the lack of differences in total Ca levels between groups MIBI(+) and MIBI(−) can be explained by the fact that patients received treatment to reduce hypercalcemia (fluids, diuretics, bisphosphonates) for varying lengths of time, with varying degrees of decrease in calcemia. The onset of the disease to its diagnosis and subsequent referral to the surgeon varied and can sometimes take several years. Hypercalcemia in PHPT develops gradually, with its level not necessarily reflecting the weight or metabolism of PA. We also note the so-called normocalcemic PHPT [40]. In such cases, radiotracer uptake may be visible during scintigraphy, which depends on the metabolism of the PA cells and not on the concentration of Ca. All these factors should be accounted for in the interpretation of the relationship between total Ca concentration and the result of the scintigraphy.

This study showed no relationship between scintigraphy results and demographic data, including age and sex, which is consistent with other studies [21,38]. However, some studies show that women have more frequent positive scintigraphy results [22].

Our findings showed that the size and mass of the PG were significant factors affecting the visualization of PG during scintigraphy (*p* < 0.01, *p* < 0.01, respectively), which is also consistent with the data from the literature [15,20,21,22,30,33]. Conversely, some studies found no relationship between the volume of the affected gland and scintigraphy results [38]. Our results indicate that small PGs may be undetectable in MIBI scintigraphy, while larger glands are easier to identify, which is important for planning surgical procedures, including MIP. However, it should be emphasized that the absence of tracer uptake in scintigraphy does not rule out the presence of disease and indicates the need for complementary imaging methods. Although our results indicate that the size of abnormal PGs affects the outcome of scintigraphy, this test should not be omitted from the diagnostic process. Small adenomas are often not visible on US, so even minimal or trace uptake in MIBI scintigraphy can be helpful to the surgeon in locating the abnormal gland and planning the extent of surgery.

The usefulness of the Wisconsin Index has been described in the literature as a tool for predicting adenoma weight [41] and for stratifying the risk of multiglandular disease [42]. In our study group, the Wisconsin Index was associated with a higher likelihood of positive MIBI scintigraphy (p = 0.02), which may indicate its potential value as a supportive preoperative marker of scintigraphic detectability in patients with PHPT; however, further validation is required.

Regarding the location of the affected PGs, our study found that adenomas were more often located in the lower PGs than in the upper PGs. However, this difference was not significant and may be helpful in the absence of visualization of the altered PGs on imaging studies. Negative scintigraphy results were observed in cases in which the adenomas were located in the upper PGs [20]. In contrast, another study showed that positive scintigraphy was significantly associated with lesion location in the lower PGs [22]. In our study, four patients showed the presence of adenomas in ≥2 PGs. After analyzing differences between single-gland disease (SGD) and polyglandular disease (PGD), one study found that preoperative Ca and iPTH levels were higher, and US and Sestamibi outcomes were more likely in SGD than in PGD [43].

The topic of PC as a cause of PHPT requires a separate discussion. PC is an extremely rare malignant tumor of the PGs (<1%) [44] that can metastasize to the bones, lungs, and liver. Non-functional PCs, although very rare, can lead to false-negative MIBI results [45,46]. In our study, one case involved a 70-year-old woman whose PC diagnosis was very challenging to establish due to the limited contribution of imaging. Another study by Cheon et al. showed that patients with PC were significantly older, had higher concentrations of PTH, and had more intense radioactivity than patients with a benign tumor [34]. The literature reports a case of PC with lung metastases in which no radioactive tracer uptake was observed in the parathyroid glands. In contrast, the lung metastases were overactive and effectively located on Tc-99m-MIBI SPECT/CT [47]. Another study also reported a false-negative (99m)Tc-Sestamibi result in PC, which was detected on 18F-FDG-PET/CT [48]. All of these rare PC cases pose a major challenge for clinicians.

Finally, we emphasize that thyroid diseases, including hyperthyroidism or thyroiditis, can also be the cause of false-negative MIBI results [49,50]. Therefore, additional SPECT/CT is preferred in these patients [51].

Although the majority of patients in our study group underwent US and MIBI SPECT/CT scintigraphy for localization of the pathological PG, it should be emphasized that F-18 choline PET/CT is currently considered the standard imaging modality for PG evaluation [52]. Recent studies directly comparing F-18 choline PET/CT with 99mTc-MIBI SPECT/CT demonstrate that FCH PET/CT often yields higher sensitivity, positive predictive value, and improved biochemical cure rates (normalization of serum Ca and PTH) than the conventional MIBI approach, especially in discordant cases and smaller or lower-activity adenomas. In particular, Kang et al. reported a sensitivity of 97.5% for FCH PET/CT versus 74.4% for 99mTc-MIBI, with higher rates of biochemical cure defined by normalization of both calcium and PTH in cases localized only by FCH PET/CT, highlighting the clinical relevance of improved detection for biochemical outcomes [53]. Likewise, Araz et al. showed FCH PET/CT’s superior diagnostic accuracy and noted correlations between SUVmax on PET and biochemical parameters such as serum PTH and Ca levels, which may reflect adenoma activity and contribute to understanding interrelations between imaging uptake, hormone secretion, and adenoma burden [54]. In our study cohort, only a small number of patients underwent this examination due to its limited availability. Moreover, our analysis was retrospective in nature and included patients who had already undergone surgical treatment. The implementation of F-18 choline PET/CT would likely have enhanced preoperative localization, thereby potentially improving surgical planning and overall treatment efficacy.

Our study has several limitations. A key limitation of this study is that imaging examinations and biochemical tests were performed in multiple external centers. Differences in imaging protocols, equipment, laboratory assays, reference ranges, and interpretation practices may have introduced measurement variability and potential bias. Although all results were reviewed and analyzed according to predefined criteria at our center, residual interinstitutional heterogeneity cannot be excluded. Additionally, the limitations of the study include its retrospective design, the lack of detailed data on disease duration and treatment, and the assessment of Ca and PTH levels only on the first postoperative day, which may have influenced the results. We did not have access to follow-up laboratory data, as patients were referred for surgery from various medical centers and subsequently returned to their primary physicians for continued care. Therefore, additional postoperative blood measurements were not available for analysis, which should be considered a limitation of this study.

## 5. Conclusions

Our study demonstrated that 99mTc-MIBI scintigraphy may yield negative results even in patients with biochemically confirmed PHPT. Imaging outcomes appear to be influenced by various factors, including PTH levels, Wisconsin index, and the size and weight of the affected PGs. A negative MIBI scan is often associated with only mildly elevated PTH levels and should not be interpreted as evidence against the presence of disease. Therefore, accurate diagnosis of PHPT requires a comprehensive evaluation that integrates biochemical, clinical, and imaging data to avoid misdiagnosis and delays in surgical referral.

## Figures and Tables

**Table 1:** Demographic, clinical, and biochemical characteristics of the study group (n = 220).

Characteristics	Results
Age (years)	
Mean (SD)	56.6 (14.2)
Gender, n (%)	
Female/male	189 (85.9%)/31 (14.1%)
Preoperative Ca total (mmol/L)	
Mean (SD)	2.8 (0.2)
Minimum/maximum	2.4/3.6
Preoperative Ca ionized (mmol/L)	
Mean (SD)	1.5 (0.1)
Postoperative Ca total (mmol/L)	
Mean (SD)	2.4 (0.2)
Postoperative Ca ionized (mmol/L)	
Mean (SD)	1.2 (0.1)
Preoperative PTH (pg/mL)	
Mean (SD)	165.1 (130.2)
Minimum/maximum	52.0/1160
Postoperative PTH (pg/mL)	
Mean (SD)	21.9 (16.6)
Normalization of total Ca after the surgery	
No/yes	21 (9.5%)/180 (81.8%)
Missing	19 (8.6%)
Normalization of PTH after surgery	
No/yes	4 (1.8%)/201 (91.4%)
Missing	15 (6.8%)
Max size of affected PG (mm)	
Mean (SD)	19.7 (9.1)
Total excised PG weight (g)	
Mean (SD)	1.8 (2.6)
Result of MIBI SPECT/CT	
Positive/Negative	164 (74.5%)/50 (22.7%)
Missing	6 (2.7%)
Result of parathyroid US	
Positive/Negative	169 (76.8%)/18 (8.2%)
Missing	33 (15.0%)
Histopathology, n (%)	
Parathyroid adenoma	208 (94.5%)
Parathyroid hyperplasia	9 (4.1%)
Parathyroid cancer	1 (0.5%)
Negative exploration	1 (0.5%)
Normal gland	1 (0.5%)
Localization	
Upper-right	21 (9.5%)
Lower-right	72 (32.7%)
Upper-left	25 (11.4%)
Lower-left	62 (28.2%)
≥2 PGs	4 (1.8%)
Ectopia	3 (1.4%)
Missing	33 (15.0%)
Localization, upper/lower	
Upper: right + left	46 (20.9%)
Lower: right + left	134 (60.9%)
≥2 PGs	4 (1.8%)
Ectopia	3 (1.4%)
Missing	33 (15.0%)
Type of exploration, n (%)	
Bilateral neck exploration	29 (13.2%)
Unilateral neck exploration	145 (65.9%)
Focused parathyroidectomy	46 (20.9%)
Symptoms of HPT disease, n (%)	
Fatigue, no/yes	179 (81.4%)/41 (18.6%)
Osteopenia/osteoporosis, no/yes	150 (68.2%)/70 (31.8%)
Kidney stone, no/yes	140 (63.6%)/80 (36.4%)
Neuropsychiatric symptoms, no/yes	207 (94.1%)/13 (5.9%)

**Table 2:** Comparison between positive and negative MIBI groups based on SPECT/CT (n = 212).

	Sestamibi Scintigraphy		
Variable	Negative MIBI (N = 49)	Positive MIBI (N = 163)	*p*-Value
Age (years)			
Mean (SD)	56.2 (14.5)	56.4 (14.1)	0.93
Sex			
F/M	45 (91.8%)/4 (8.2%)	136 (83.4%)/27 (16.6%)	0.14
Preoperative Ca total (mmol/L)			
Mean (SD)	2.8 (0.2)	2.8 (0.2)	0.59
Minimum/maximum	2.4/3.2	2.4/3.6	
Preoperative Ca ionized (mmol/L)			
Mean (SD)	1.5 (0.1)	1.5 (0.1)	0.58
Postoperative Ca total (mmol/L)			
Mean (SD)	2.4 (0.2)	2.4 (0.2)	0.64
Postoperative Ca ionized (mmol/L)			
Mean (SD)	1.3 (0.1)	1.2 (0.1)	0.60
Postoperative Ca total normalization			
No	8 (16.0%)	12 (7.4%)	0.09
Yes	38 (77.5%)	135 (82.8%)	
Missing	3 (6.1%)	16 (9.8%)	
Preoperative PTH (pg/mL)			
Mean (SD)	127.7 (59.4)	177.3 (144.7)	<0.01
Minimum/maximum	60. 0/1160.0	52.0/345.0	
Postoperative PTH (pg/mL)			
Mean (SD)	21.1 (14.5)	22.4 (17.3)	0.78
Postoperative PTH normalization			
No	0 (0.0%)	4 (2.4%)	0.58
Yes	47 (95.9%)	149 (91.4%)	
Missing	2 (4.1%)	10 (6.1%)	
Wisconsin index (preoperative Ca × PTH)			
Mean (SD)	355.4 (172.4)	507.0 (428.7)	<0.01
Max size of PG (mm)			
Mean (SD)	13.9 (5.3)	21.5 (9.2)	<0.01
Excised PG weight (g)			
Mean (SD)	1.1 (2.8)	2.1 (2.6)	<0.01
Preoperative USG			
Negative	8 (16.3%)	9 (5.5%)	0.03
Positive	34 (69.4%)	130 (79.6%)	
Missing	7 (14.3%)	24 (14.7%)	
Localization			
Upper-right	1 (2.0%)	18 (11.0%)	0.53
Lower-right	12 (24.5%)	58 (35.6%)	
Upper-left	4 (8.2%)	21 (12.9%)	
Lower-left	13 (26.5%)	49 (30.1%)	
≥2 PGs	0 (0.0%)	4 (2.5%)	
Ectopia	0 (0.0%)	3 (1.8%)	
Missing	19 (38.8%)	10 (6.1%)	
Localization			
Upper: right + left	5 (16.7%)	39 (26.7%)	0.25 *
Lower: right + left	25 (83.3%)	107 (73.3%)	
Histopathology			
Parathyroid adenoma	45 (91.8%)	157 (96.3%)	0.14
Parathyroid hyperplasia	3 (6.1%)	6 (3.7%)	
Parathyroid cancer	1 (2.0%)	0 (0.0%)	
Type of surgery			
Reoperation	3 (6.1%)	3 (1.8%)	0.14
Primary operation	46 (93.9%)	160 (98.2%)	

* Chi-square test performed only for comparisons between PPG + PLG and PPD + PLD groups versus scintigraphy groups.

**Table 3:** Univariate (crude) and multivariate (adjusted) logistic regression models (model 1 and model 2) assessing predictors of positive scintigraphy. Odds ratios (OR) and 95% confidence intervals (CI) are presented.

Predictors	Crude OR (95% CI)	*p*-Value	Adjusted OR (95% CI)	*p*-Value	Adjusted OR (95% CI)	*p*-Value
			(Model 1) ᵟ		(Model 2) ᵠ	
Sex (M vs. F)	2.23 (0.74–6.73)	0.15				
Age (years) *	1.05 (0.76–1.45)	0.76				
Ca total (mmol/L) *	1.15 (0.82–1.62)	0.40	0.96 (0.66–1.41)	0.85	0.89 (0.58–1.37)	0.60
PTH (pg/mL) *	2.65 (1.19–5.89)	0.02	2.62 (1.14–5.99)	0.02	2.47 (0.95–6.41)	0.06
PG max size (mm) *	5.94 (2.78–12.68)	<0.01			5.87 (2.65–13.02)	<0.01

* Variable standardized (z-scores) before inclusion in the model to facilitate interpretation and comparability of ORs across predictors; ᵟ model with S-calcium and PTH only; ᵠ model with preoperative predictors; Model 1 fit statistics: Hosmer–Lemeshow χ^2^ = 8.2, *p* = 0.4; AUC = 0.64 (SE = 0.05); Cox–Snell R^2^ = 0.04; Nagelkerke R^2^ = 0.06; Model 2 fit statistics: Hosmer–Lemeshow χ^2^ = 10.5, *p* = 0.23; AUC = 0.81 (SE = 0.04); Cox–Snell R^2^ = 0.19; Nagelkerke R^2^ = 0.30.

**Table 4:** Percentage of patients with positive scintigraphy results exceeding combined thresholds of total calcium (2.60–3.20 mmol/L) and PTH (90–170 pg/mL) in a 13 × 17 grid analysis, with a grayscale gradient from white (lowest) to dark gray (highest values).

PTH	90	95	100	105	110	115	120	125	130	135	140	145	150	155	160	165	170
Total Calcium																	
2.60	80.1	81.0	80.1	80.6	80.3	81.4	81.1	80.6	82.3	86.0	86.6	86.3	85.1	85.7	87.3	88.3	87.0
2.65	82.9	84.0	83.2	83.2	83.2	84.5	83.7	83.5	85.4	87.7	88.5	88.2	87.1	86.8	88.5	88.1	86.8
2.70	82.2	82.7	81.9	82.4	82.8	84.4	83.7	83.8	85.9	88.9	89.9	89.6	88.7	88.5	91.2	90.9	89.8
2.75	79.6	79.6	78.9	79.3	80.5	82.3	81.6	81.7	84.1	87.5	88.5	88.1	87.3	87.0	90.0	89.6	88.4
2.80	78.9	79.2	78.6	79.4	81.5	82.5	81.7	82.5	85.5	86.5	87.8	87.5	86.7	86.4	90.0	89.7	88.9
2.85	76.3	77.6	76.8	78.2	81.1	82.4	81.3	80.9	84.4	83.7	85.0	84.6	83.8	83.8	88.2	87.9	87.1
2.90	76.1	77.8	77.3	79.1	82.9	85.0	84.2	84.2	86.5	85.7	87.9	87.5	86.7	86.7	89.3	88.9	88.9
2.95	80.6	80.6	80.0	82.4	87.5	87.5	86.7	86.7	86.7	85.7	88.5	88.0	87.5	87.5	90.9	90.9	90.9
3.00	81.0	81.0	81.0	81.0	89.5	89.5	88.2	88.2	88.2	87.5	86.7	86.7	86.7	86.7	92.3	92.3	92.3
3.05	83.3	83.3	83.3	83.3	88.2	88.2	86.7	86.7	86.7	85.7	84.6	84.6	84.6	84.6	90.9	90.9	90.9
3.10	85.7	85.7	85.7	85.7	92.3	92.3	91.7	91.7	91.7	90.9	90.0	90.0	90.0	90.0	100.0	100.0	100.0
3.15	90.9	90.9	90.9	90.9	100.0	100.0	100.0	100.0	100.0	100.0	100.0	100.0	100.0	100.0	100.0	100.0	100.0

Legend: Grayscale shades indicate the percentage of patients with positive scintigraphy results among those exceeding both calcium and PTH thresholds. White, lowest values; dark gray, highest; intermediate shades of gray, intermediate percentages.

**Table 5:** Characteristics of biochemical findings in patients with negative MIBI.

Parameters	Before Surgery	After Surgery	*p*-Value *
Calcium total (mmol/L)			
Mean (SD)	2.8 (0.2)	2.4 (0.2)	<0.001
Calcium ionized (mmol/L)			
Mean (SD)	1.5 (0.1)	1.2 (0.1)	<0.001
PTH (pg/mL)			
Mean (SD)	128.9 (59.4)	20.3 (13.6)	<0.001
Histopathology	PA: 45 patients (91.8%)		
	PH: 3 patients (6.1%)		
	PC: 1 patient (2.0%)		
PG localization	Upper-right: 1 (2.0%)		
	Lower-right: 12 (24.5%)		
	Upper-left: 4 (8.2%)		
	Lower-left: 13 (26.5%)		
	Missing date: 19 (38.8%)		

* Wilcoxon signed-rank test for paired samples.

## Data Availability

The original contributions presented in the study are included in the article. Further inquiries can be directed to the corresponding authors.
